# Comparative Treatment Response to Intermittent Theta Burst Stimulation in Long COVID-Associated Depression Versus Major Depressive Disorder: A Propensity Score-Matched Retrospective Cohort Study

**DOI:** 10.3390/biomedicines14081872

**Published:** 2026-08-21

**Authors:** Yoshihiro Noda, Ryota Osawa, Yuya Takeda, Ryosuke Kitahata

**Affiliations:** 1 Department of Psychiatry, International University of Health and Welfare, Mita Hospital, Tokyo 108-8329, Japan; 2 Tokyo Yokohama TMS Clinic, Minato-Tokyo Branch, Tokyo 108-0023, Japan; 3 Tokyo Yokohama TMS Clinic, Kosugi-Kanagawa Branch, Kawasaki 211-0063, Japan; 4 Shinjuku-Yoyogi Mental Lab Clinic, Tokyo 151-0051, Japan

**Keywords:** COVID-19, depressive disorder, transcranial magnetic stimulation, theta burst stimulation, propensity score matching

## Abstract

**Background:** Long COVID has emerged as a global health challenge characterized by persistent neuropsychiatric symptoms, including depression, fatigue, and cognitive impairment. Growing evidence indicates that sustained neuroinflammation, endothelial dysfunction, and fronto-limbic network disruption may underlie these symptoms, representing pathophysiological features distinct from major depressive disorder (MDD). Although repetitive transcranial magnetic stimulation (rTMS) is an established treatment for MDD, its therapeutic efficacy in Long COVID-associated depression remains uncertain. **Methods:** Long COVID was defined as laboratory-confirmed SARS-CoV-2 infection followed by persistent neuropsychiatric symptoms lasting ≥3 months, including cognitive impairment (“brain fog”), fatigue, and new-onset depressive symptoms. We conducted a retrospective registry-based cohort study using real-world clinical data from two TMS clinics in Tokyo (May 2022–April 2026). Forty-four medication-free patients with Long COVID-associated MDD were compared with eighty-eight propensity score–matched patients with primary MDD (1:2 nearest-neighbor matching based on age, sex, and baseline Montgomery–Åsberg Depression Rating Scale (MADRS)). All participants received left dorsolateral prefrontal cortex intermittent theta burst stimulation (iTBS). Treatment outcomes were evaluated using MADRS and 17-item Hamilton Depression Rating Scale (HAM-D_17_) improvement rates, HAM-D_17_ response, and remission. Statistically adjusted analyses (inverse probability of treatment weighting (IPTW) and doubly robust estimation) were used to reduce measured confounding; however, residual unmeasured confounding cannot be excluded due to the retrospective observational design. **Results:** Long COVID patients showed significantly attenuated antidepressant response. Improvement rates were markedly lower for MADRS (38.4% vs. 61.5%) and HAM-D_17_ (36.7% vs. 58.4%). IPTW and doubly robust models demonstrated large adjusted percentage-point differences (MADRS: −23.0 percentage points; HAM-D_17_: −21.8 percentage points; both *p* < 0.001). While HAM-D_17_ response did not differ significantly, remission rates were substantially reduced (27.3% vs. 61.4%). **Conclusions:** These findings indicate an adjusted association suggesting reduced rTMS responsiveness in Long COVID-associated depression. Given the retrospective observational design and the possibility of residual unmeasured confounding, the results should be interpreted as associations rather than evidence of a causal effect.

## 1. Introduction

Persistent neuropsychiatric symptoms such as depression, chronic fatigue, and cognitive impairments, including deficits in attention and memory commonly referred to as brain fog, may continue long after the resolution of acute COVID-19 symptoms. This condition, known as Long COVID, has increasingly been recognized as a global public health challenge. These symptoms can persist for several months to years following infection and have been widely reported to substantially impair daily functioning, occupational performance, and social participation [1,2,3,4,5].

Accumulating evidence suggests that the neuropsychiatric manifestations of Long COVID arise from a complex interplay of multiple biological factors, including sustained neuroinflammation, prolonged immune activation, vascular endothelial dysfunction, disruptions in functional brain networks, and metabolic abnormalities. These mechanisms likely reflect a pathophysiological profile distinct from that of conventional major depressive disorder (MDD). Notably, SARS-CoV-2 infection has been shown to increase blood–brain barrier permeability and induce neuroinflammatory responses, leading to impaired functional connectivity within large-scale brain networks. Such alterations may underlie the depressive symptoms and cognitive deficits observed in individuals with Long COVID [6,7,8,9]. However, it is important to note that these mechanistic hypotheses are derived from prior biological and neuroimaging studies and were not directly assessed in the present clinical dataset; therefore, the subsequent discussion regarding potential links between Long COVID pathophysiology and rTMS responsiveness should be interpreted as a theoretically grounded inference rather than a direct empirical conclusion drawn from the current sample.

However, therapeutic options for the neuropsychiatric manifestations of Long COVID remain limited, and effective interventions for depressive symptoms and cognitive impairment have yet to be firmly established [10,11,12,13]. To address this clinical gap, we previously conducted an observational real-world study in 2023 involving 23 patients with Long COVID-associated MDD who received repetitive transcranial magnetic stimulation (rTMS) in routine clinical practice [14,15]. In that study, rTMS demonstrated potential clinical utility, with mean Montgomery–Åsberg Depression Rating Scale (MADRS) scores improving from approximately 21 to 10, suggesting a substantial antidepressant effect. Nevertheless, because the prior investigation lacked a control group and did not adjust for confounding, the extent to which Long COVID–specific factors influenced treatment responsiveness remained unclear.

Although depressive symptoms associated with Long COVID often resemble those observed in primary major depressive disorder (MDD), accumulating evidence indicates that COVID-19–specific pathophysiological processes, such as persistent neuroinflammation, immune dysregulation, functional disruption of large-scale brain networks, and the frequent co-occurrence of chronic fatigue, may substantially influence treatment responsiveness [16,17,18]. Indeed, several studies have reported increased incidence of depressive symptoms following SARS-CoV-2 infection, accompanied by high rates of cognitive impairment and fatigue, suggesting that the clinical phenotype of Long COVID–related depression differs meaningfully from that of conventional MDD [19,20,21]. These clinical and biological distinctions raise a critical question as to whether depressive symptoms emerging in the context of Long COVID exhibit comparable responsiveness to rTMS as primary MDD, or whether COVID-19–related neurobiological alterations confer a distinct and potentially more treatment-resistant phenotype.

This study was designed as a retrospective cohort analysis extending our previous observational case-series investigation conducted in 2023 [14]. The aim was to directly compare rTMS treatment responsiveness between patients with Long COVID-associated MDD and those with primary MDD. Specifically, we analyzed clinical registry data comprising 44 patients with Long COVID–related depression and 250 patients with primary MDD. Using age, sex, and baseline MADRS scores as covariates, we performed 1:2 propensity score matching to select 88 matched MDD controls. Long COVID was defined as laboratory-confirmed SARS-CoV-2 infection followed by persistent neuropsychiatric symptoms lasting ≥3 months, including cognitive impairment (“brain fog”), fatigue, and new-onset depressive or anxiety symptoms. All participants in the Long COVID group met DSM-5 criteria for major depressive disorder; anxiety symptoms alone were not sufficient for inclusion.

In this study, propensity score matching was performed using a minimal set of core demographic and baseline severity variables to establish fundamental comparability between groups, whereas subsequent inverse probability of treatment weighting (IPTW) and doubly robust estimation incorporated additional treatment-related covariates to more comprehensively mitigate residual confounding.

Based on prior clinical experience and earlier observational findings, we post-hoc hypothesized that patients with Long COVID-associated MDD would demonstrate lower therapeutic responsiveness to rTMS than those with primary MDD, such that both depressive symptom improvement (MADRS and 17-item Hamilton Depression Rating Scale (HAM-D_17_)) and remission rates would be significantly reduced in the Long COVID group. This hypothesis was exploratory and intended to guide comparative analysis rather than to infer causal modification of treatment efficacy.

## 2. Materials and Methods

### 2.1. Study Design and Participants

This study was conducted as a retrospective registry-based cohort investigation using real-world clinical data [22] from two TMS clinics in the Tokyo metropolitan area between May 2022 and April 2026. At both sites, rTMS treatment was delivered within a clinical framework grounded in shared decision making [23], which guided diagnostic evaluation and treatment planning throughout the course of care. Because the study relied on real-world clinical data rather than a standardized trial protocol, treatment parameters and clinical decision-making processes were not experimentally controlled; therefore, the analytic strategy was designed to explicitly account for potential heterogeneity in treatment delivery through covariate adjustment and propensity-score–based weighting methods.

Two patient groups were included in the analysis:

1. Long COVID-associated MDD group: Patients presenting with depressive symptoms associated with Long COVID who met DSM-5 criteria for MDD and received rTMS treatment.
2. Primary MDD group: Patients without Long COVID who were diagnosed with primary MDD and received rTMS treatment.

Long COVID was defined according to WHO criteria as laboratory-confirmed SARS-CoV-2 infection followed by persistent physical or neuropsychiatric symptoms lasting ≥3 months, including chronic fatigue, myalgia, cognitive impairment (“brain fog”), insomnia, headache, and newly developed depressive or anxiety symptoms. In this cohort, all patients in the Long COVID group had non-severe acute COVID-19 that did not require hospitalization. The duration of Long COVID symptoms ranged from approximately 3 months to 2.5 years. Fatigue and cognitive impairment were assessed qualitatively during routine clinical interviews, as no standardized scales were administered.

To construct a comparable control cohort, propensity scores were estimated using a logistic regression model incorporating age, sex, and baseline MADRS severity. A 1:2 nearest-neighbor matching procedure was then applied to identify 88 matched MDD patients from a pool of 250 registry cases [24,25]. The propensity score model was intentionally limited to core demographic (age, sex, and baseline MADRS) and severity variables due to sample size constraints and the absence of structured data for several clinically relevant predictors (e.g., Long COVID duration, fatigue severity, cognitive impairment, medical comorbidities). Because these variables were unavailable in the registry dataset, they could not be incorporated into the propensity score model, and residual unmeasured confounding cannot be excluded.

Furthermore, to eliminate pharmacological confounding, the analytic sample was restricted to individuals who were not taking any psychiatric medications, including antidepressants, at the time of rTMS treatment.

**Figure 1:**
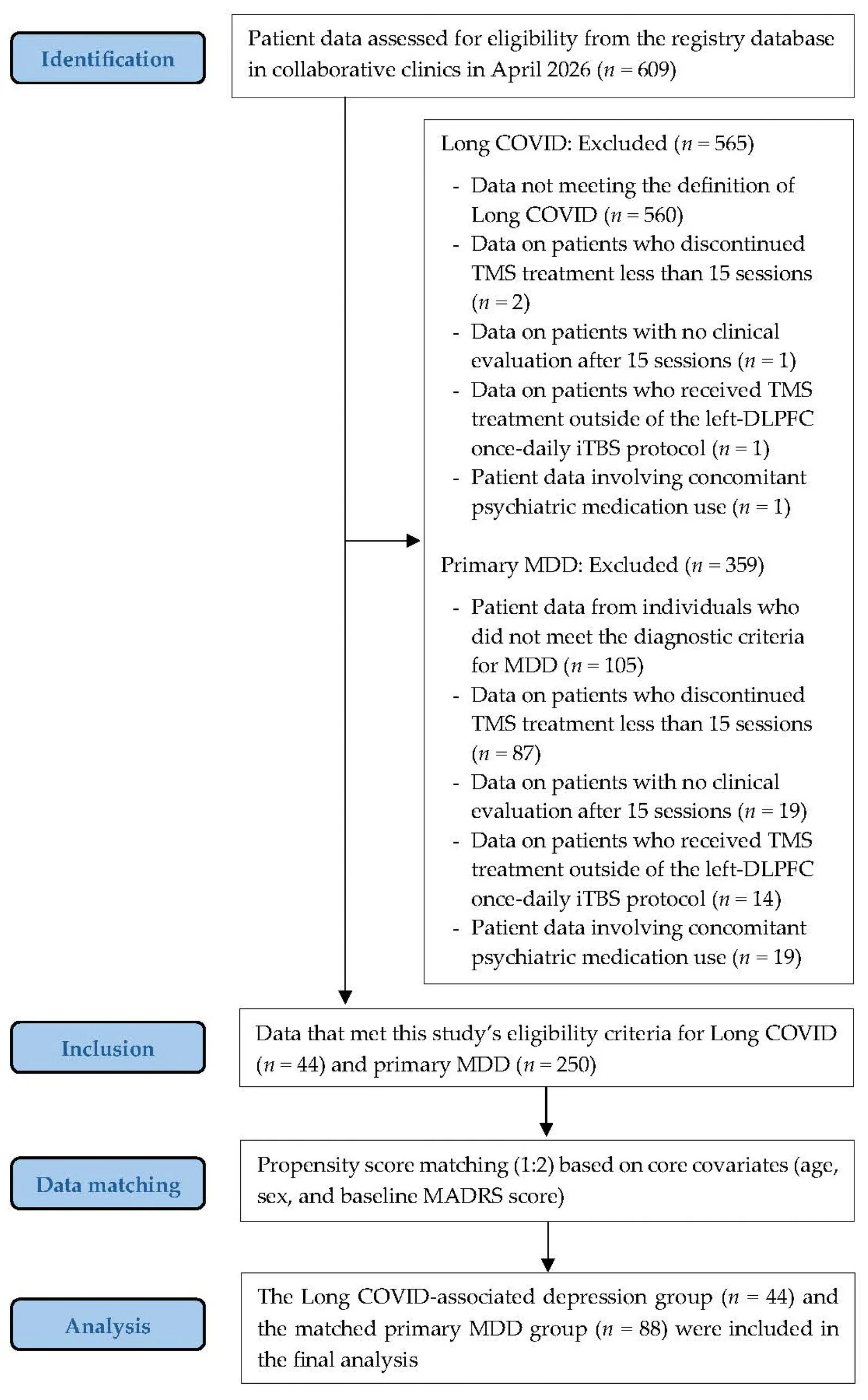
STROBE-style participant flow diagram illustrating the number of patients screened, excluded, matched, and included in the final analyses.

The overall screening, exclusion, and matching process is illustrated in Figure 1**,** which presents a STROBE-style participant flow diagram detailing the number of patients assessed, excluded, matched, and included in the final analysis.

### 2.2. Inclusion and Exclusion Criteria

Major depressive disorder (MDD) was diagnosed according to DSM-5 criteria based on comprehensive, non-structured clinical interviews conducted by board-certified psychiatrists. Comorbid anxiety symptoms were evaluated during the same clinical interview, and only patients who developed depressive or anxiety symptoms for the first time after confirmed SARS-CoV-2 infection were eligible for inclusion in the Long COVID group.

Exclusion criteria included:

- Bipolar disorder, psychotic disorders, substance use disorders, or primary sleep disorders
- Unstable medical or neurological conditions
- History of epilepsy or convulsive seizures
- Contraindications to TMS (e.g., metallic implants, pacemakers)
- Presence of active major psychiatric disorders prior to SARS-CoV-2 infection (for the Long COVID group)

These criteria ensured that the cohort represented a clinically homogeneous group appropriate for rTMS intervention.

### 2.3. TMS Treatment Protocol

rTMS treatment was administered within routine clinical practice using a shared decision-making framework. The primary protocol consisted of high-dose intermittent theta burst stimulation (iTBS) [26] targeting the left dorsolateral prefrontal cortex (DLPFC) [27]. Each session delivered 1200 pulses over approximately six minutes, with treatments provided two to three times per week for a total of 20 to 30 sessions. Stimulation intensity was generally set at 120% of the resting motor threshold (RMT), although adjustments within the range of 100–120% were made depending on individual tolerability. All treatments were performed using a MagPro R30 stimulator equipped with a B70-Cool coil (MagVenture, Farum, Denmark), and the left DLPFC target was localized using the Beam F3 method [28,29].

Because treatment parameters were determined through shared decision-making [23] rather than strict protocolization, stimulation intensity and session count varied across patients and were therefore incorporated as covariates in weighting and regression models to mitigate confounding arising from individualized treatment variation.

### 2.4. Outcome Measures

The primary continuous outcomes were the percentage change from baseline in MADRS [30] and HAM-D_17_ [31] scores. Binary outcomes included HAM-D_17_ response, defined as a reduction of 50% or greater from baseline, and HAM-D_17_ remission, defined as a post-treatment HAM-D_17_ score of 7 or below. Percentage change outcomes were selected to facilitate comparability across individuals with differing baseline severity, and remission was included as a clinically stringent endpoint that may be more sensitive to pathophysiological differences between Long COVID and primary MDD.

### 2.5. Statistical Analyses

Statistical analyses were conducted using Python (version 3.10) and R (version 4.2). Covariates included age, sex, baseline MADRS, baseline HAM-D_17_, stimulation intensity (% RMT), total number of treatment sessions, and treatment facility (included as a fixed effect to account for site-level variation). Age, sex, and baseline MADRS were included in the propensity score model for matching and IPTW. Baseline HAM-D17, stimulation intensity, number of sessions, and treatment facility were not part of the propensity score but were incorporated only in subsequent regression adjustment (IPTW-weighted and doubly robust models).

Propensity score matching was performed using logistic regression with age, sex, and baseline MADRS as covariates. Covariate balance was assessed using standardized mean differences (SMDs). A Love plot was used to visualize covariate balance before and after matching. Although stimulation intensity and session number exhibited minor residual imbalance (SMDs > 0.1), these variables were subsequently adjusted using inverse probability of treatment weighting (IPTW) and regression models. Based on these scores, 1:2 nearest-neighbor matching was performed, yielding 88 matched MDD patients for the 44 Long COVID patients. For the matched dataset, inverse probability of treatment weights were calculated using the following formula:

$$w_{i}=\frac{LC_{i}}{PS_{i}}+\frac{1-LC_{i}}{1-PS_{i}}.$$

These weights were applied in weighted least squares regression models to compare improvement rates between groups [32,33]. IPTW was applied after matching to further reduce residual confounding and to approximate a pseudo-population in which covariates are independent of treatment assignment. Doubly robust estimation was implemented by combining IPTW with regression adjustment for treatment facility. Because this study is retrospective and observational, these methods can reduce, but cannot eliminate, unmeasured confounding [34,35]; therefore, all results represent adjusted associations rather than causal effects.

Overlap of propensity score distributions was examined to confirm adequate common support [36,37,38] between the Long COVID and MDD groups.

To assess the robustness of the propensity score–matched comparisons, we conducted sensitivity analyses using three alternative matching specifications: (i) nearest-neighbor matching without replacement, (ii) caliper-restricted matching with a caliper width of 0.20 SD of the logit of the propensity score, and (iii) matching models that additionally included baseline HAM-D_17_ as a covariate. These specifications were selected to evaluate the stability of covariate balance and treatment-effect estimates under different matching constraints.

### 2.6. Ethical Consideration

This retrospective cohort study utilized registry-based TMS data collected during routine clinical care and was reviewed and approved by the Ethics Committee of ITO Yoyogi Mental Clinic (approval number: RKK319). All analyses were conducted using anonymized data, with strict adherence to procedures ensuring the protection of personal information.

## 3. Results

### 3.1. Baseline Characteristics After Propensity Score Matching

Following 1:2 propensity score matching, all major covariates: age, sex, baseline MADRS, and baseline HAM-D_17_, achieved standardized mean differences (SMDs) below 0.1, indicating that the Long COVID and MDD groups were well balanced with respect to key background characteristics. Baseline demographic and clinical characteristics for both groups are summarized in Table 1.

A total of 609 patients were assessed for eligibility. After applying inclusion and exclusion criteria, 44 patients with Long COVID-associated depression and 250 patients with primary MDD were eligible. Propensity score matching (1:2 nearest neighbor matching based on age, sex, and baseline MADRS) yielded 44 Long COVID patients and 88 matched MDD patients for the final analysis.

The Love plot (Figure S1) demonstrated substantial improvement in covariate balance after matching, with core demographic and baseline severity variables achieving SMD values below 0.1. However, stimulation intensity and the number of treatment sessions exhibited minor residual imbalance (SMDs of 0.12 and 0.14), exceeding the prespecified threshold of 0.10. These variables were therefore adjusted in subsequent IPTW and regression models.

Importantly, no covariate exhibited an SMD exceeding 0.15, and visual inspection of covariate distributions confirmed that residual imbalance was modest.

The propensity score overlap plot demonstrated broad distributional overlap between the Long COVID and MDD groups, confirming adequate common support (Figure S2). The plot indicates that comparisons were conducted within a shared covariate space, although residual unmeasured confounding cannot be excluded due to the observational design.

### 3.2. Continuous Outcomes: MADRS and HAM-D_17_ Improvement Rates

After propensity score matching, the mean improvement rates for depressive symptoms differed markedly between groups. The Long COVID group showed a mean MADRS improvement of 38.4% ± 22.1%, whereas the MDD group demonstrated a substantially greater improvement of 61.5% ± 20.3%. A similar pattern was observed for HAM-D_17_ scores, with the Long COVID group improving by 36.7% ± 21.8% compared with 58.4% ± 19.9% in the MDD group. These descriptive differences represent large adjusted associations, reflecting substantial group-level differences in improvement rates.

Weighted least squares regression using IPTW further quantified these differences. Long COVID status was associated with a −23.0 percentage-point adjusted difference in MADRS improvement (*p* < 0.001) and a −21.8 percentage-point adjusted difference in HAM-D_17_ improvement (*p* < 0.001). These coefficients represent percentage-point differences in improvement rates, not raw score differences.

Doubly robust estimation yielded nearly identical results, confirming the robustness of these findings. For MADRS improvement, the coefficient was −23.011 percentage points (95% CI: −30.107 to −15.915, *p* < 0.001), and for HAM-D_17_ improvement, the coefficient was −21.768 percentage points (95% CI: −29.035 to −14.500, *p* < 0.001). The consistency across matched comparisons, IPTW, and doubly robust models indicates a stable adjusted association between Long COVID status and reduced rTMS responsiveness.

To further evaluate the robustness of these findings, additional sensitivity analyses were conducted using alternative matching specifications, including nearest-neighbor matching without replacement, caliper-restricted matching (caliper = 0.20 SD of the logit of the propensity score), and matching models incorporating baseline HAM-D_17_ as an additional covariate. Across all specifications, the adjusted percentage-point differences in improvement rates consistently indicated reduced responsiveness in the Long COVID group. The estimated differences ranged from −18.57 to −9.97 percentage points for MADRS and −17.18 to −11.83 percentage points for HAM-D_17_, demonstrating stability of the findings under varying matching constraints. Detailed results are provided in Table S1.

### 3.3. Binary Outcomes: Response and Remission

For HAM-D_17_ response (≥50% improvement), 31.8% of patients in the Long COVID group achieved response compared with 46.6% in the MDD group. Logistic regression indicated that Long COVID status was associated with an odds ratio of 0.58 (coefficient = −0.539), although this difference did not reach statistical significance (*p* = 0.176). This suggests that partial symptomatic improvement may still occur in Long COVID, although the adjusted association did not reach statistical significance.

In contrast, remission outcomes showed a pronounced and statistically significant group difference. HAM-D_17_ remission (HAM-D_17_ ≤ 7) was achieved by 27.3% of Long COVID patients compared with 61.4% of MDD patients. Logistic regression revealed a coefficient of −1.225 and an OR of 0.29 (*p* = 0.002), indicating that the likelihood of achieving remission was approximately 71% lower in the Long COVID group. This adjusted association suggests that Long COVID may be linked to reduced probability of achieving full symptomatic remission following rTMS.

### 3.4. Summary of Key Findings

Overall, propensity score matching successfully balanced major covariates, enabling an adjusted comparative analysis between groups. Both IPTW and doubly robust analyses consistently demonstrated significantly lower MADRS and HAM-D_17_ improvement rates in the Long COVID group, with adjusted percentage-point differences of approximately −22 to −23 points.

Although response rates did not differ significantly, remission rates were markedly reduced among Long COVID patients (OR ≈ 0.29).

Across all analytic approaches, the results consistently indicated an adjusted association between Long COVID status and diminished rTMS treatment responsiveness.

## 4. Discussion

This study compared patients with Long COVID-associated depressive symptoms to those with primary MDD and examined differences in therapeutic responsiveness to rTMS using a retrospective registry-based cohort design. Through 1:2 propensity score matching based on age, sex, and baseline depressive severity, combined with statistically adjusted analyses incorporating IPTW and doubly robust estimation, we found that the Long COVID group exhibited significantly lower improvement rates in both MADRS and HAM-D_17_ scores compared with the matched MDD group. Moreover, remission rates were markedly reduced among patients with Long COVID. These findings represent adjusted associations rather than causal effects, given the retrospective observational design and the possibility of residual unmeasured confounding. The consistency of these findings across multiple analytic approaches strengthens the interpretation that Long COVID status is associated with reduced rTMS responsiveness, although causality cannot be inferred.

### 4.1. Clinical Implications of Reduced Treatment Responsiveness in Long COVID

The magnitude of the differences observed in this study suggests clinically relevant group-level differences. The Long COVID group showed markedly attenuated symptom improvement, with mean reductions of approximately 23 percentage points in MADRS improvement and 22 percentage points in HAM-D_17_ improvement, representing substantial deficits in therapeutic response. Remission rates were also considerably lower in the Long COVID group (27.3%) compared with the MDD group (61.4%).

The magnitude of these adjusted differences indicates a notable attenuation of treatment responsiveness in patients with Long COVID-associated depression.

Although improvement was evident in both groups, the substantially lower percentage-point reductions in MADRS and HAM-D17 scores among Long COVID patients suggest that depressive symptoms in this population may be less amenable to normalization through rTMS. Because percentage-change metrics reflect proportional improvement rather than absolute score reductions, their clinical interpretation is best grounded in the overall pattern of response rather than compared directly with thresholds derived from absolute scale-point changes. Within this framework, the consistently attenuated improvement and markedly lower remission rates observed in the Long COVID group underscore the clinical relevance of these findings. In particular, the divergence between response and remission outcomes, where partial improvement may still occur but full symptomatic normalization is substantially less likely, highlights a pattern suggesting that Long COVID-associated depression may involve disruptions in neurobiological processes required for complete recovery. However, these interpretations remain speculative and are not directly supported by mechanistic data from this study.

### 4.2. Comparison with Prior Research: Long COVID–Related Depression as a Distinct Clinical Phenotype

Although depressive symptoms in Long COVID may appear clinically similar to those observed in MDD, emerging evidence suggests that COVID-19–related biological abnormalities may contribute to symptom persistence and treatment resistance. Prior studies have proposed mechanisms such as persistent immune activation, neuroinflammation [39,40], endothelial dysfunction, mitochondrial impairment, and functional network disruption [41]. It is important to emphasize that these mechanisms were not directly evaluated in the present dataset and should be interpreted as hypothetical explanatory frameworks rather than conclusions derived from our findings.

Our results align with earlier reports indicating that Long COVID is frequently accompanied by cognitive impairment, chronic fatigue, and neuropsychiatric symptoms that may reflect a distinct clinical phenotype. The present findings suggest an association between Long COVID status and reduced rTMS responsiveness, consistent with the possibility that COVID-19–related biological alterations may influence treatment outcomes.

Nevertheless, the observational nature of this study precludes determining whether Long COVID itself modifies rTMS efficacy.

### 4.3. Positioning of the Present Study Within the Existing Literature

A previous observational study reported the potential effectiveness of rTMS in patients with Long COVID [14]; however, it was based on an open-label case series without a control group and lacked adjustment for confounding factors. The present study extends this earlier work by directly comparing treatment outcomes between Long COVID-associated depression and primary MDD using statistically adjusted comparative methods [35,42,43].

Specifically, we applied 1:2 propensity score matching, inverse probability of treatment weighting, doubly robust estimation, and logistic regression analyses for response and remission outcomes. By integrating these complementary analytic approaches, the present study reduces model dependence and provides a more robust adjusted comparison of rTMS responsiveness between groups.

Although these methods strengthen the statistical rigor of the analysis, they cannot eliminate residual unmeasured confounding, and therefore the findings should be interpreted as adjusted associations rather than causal effects.

This study represents one of the earliest statistically adjusted comparative evaluations of rTMS treatment response in Long COVID, addressing a critical gap in the existing literature and providing hypothesis-generating evidence that Long COVID may be associated with reduced antidepressant treatment responsiveness.

### 4.4. Methodological Considerations and Residual Confounding

Several methodological considerations warrant discussion. First, although propensity score matching successfully balanced core demographic and severity variables, important clinical predictors of antidepressant response, such as Long COVID duration, fatigue severity, and cognitive impairment, were not available in the structured registry dataset and therefore could not be included in the propensity score model. These factors may influence treatment responsiveness and represent potential sources of residual confounding. Second, stimulation intensity and the number of treatment sessions exhibited minor residual imbalance (SMDs of 0.12 and 0.14). Although these variables were adjusted using IPTW and regression models, residual variability in treatment delivery may still have contributed to the observed differences. Third, because all participants received iTBS and no untreated or alternative-treatment control group was included, the study cannot determine whether Long COVID modifies the intrinsic efficacy of iTBS relative to no treatment or other interventions.

### 4.5. Selection Bias and Generalizability

This study used registry data from two specialized TMS clinics in Tokyo. Patients seeking care at specialized neuromodulation centers may differ from broader clinical populations in terms of symptom severity, treatment expectations, or socioeconomic factors. Therefore, generalizability to other settings, including primary care, general psychiatry clinics, or different healthcare systems, may be limited.

### 4.6. Future Directions

Future research should include prospective double-blind randomized controlled trials [44] and incorporate neurophysiological measures [45,46] such as EEG, TMS–EEG, and functional MRI, as well as biomarkers reflecting inflammation, metabolism, and cerebral perfusion [47]. Such multimodal approaches will be critical for elucidating the biological mechanisms underlying reduced treatment responsiveness in Long COVID and for identifying potential adjunctive interventions, such as anti-inflammatory agents, metabolic enhancers, or perfusion-targeting therapies, which may augment rTMS efficacy in this population [48,49].

## 5. Conclusions

This retrospective propensity score–matched cohort study compared rTMS treatment outcomes between patients with Long COVID-associated depression and those with primary MDD. Across matched analyses, IPTW, and doubly robust estimation, patients with Long COVID consistently exhibited lower improvement rates in both MADRS and HAM-D_17_ scores, as well as substantially reduced remission rates. These findings indicate an adjusted association suggesting diminished rTMS responsiveness in Long COVID-associated depression.

Because this study is retrospective and observational, and because several clinically relevant variables could not be incorporated into the propensity score model, residual unmeasured confounding cannot be excluded. Therefore, the present results should be interpreted as adjusted associations rather than evidence of a causal effect or causal modification of rTMS efficacy.

These findings are hypothesis-generating and may help inform the design of future prospective, controlled studies aimed at clarifying whether Long COVID represents a distinct treatment-response phenotype requiring tailored neuromodulation strategies.

## Figures and Tables

**Table 1:** Baseline Demographic and Clinical Characteristics After Propensity Score Matching

Characteristic	MDD with Long COVID (*n* = 44)	Primary MDD (*n* = 88)	SMD
Age, years (mean ± SD)	38.9 ± 12.4	39.1 ± 11.8	0.02
Sex, female (%)	25 (56.8%)	50 (56.8%)	<0.01
MADRS (baseline)	21.4 ± 6.2	21.1 ± 6.0	0.04
HAM-D_17_ (baseline)	17.8 ± 5.4	17.5 ± 5.1	0.05
Stimulation intensity (% RMT)	113.4 ± 8.1	115.9 ± 7.4	0.12
Number of sessions	24.1 ± 4.8	25.3 ± 4.6	0.14

After propensity score matching, all major covariates (age, sex, and baseline MADRS/HAM-D_17_) achieved standardized mean differences (SMDs) below 0.1, indicating good balance between the Long COVID and MDD groups. Although stimulation intensity and the number of treatment sessions showed SMD values slightly above 0.1, both were substantially improved compared with pre-matching values and remained within an acceptable range for analysis. **Note:** No missing data were present for baseline variables. Standardized Mean Difference (SMD) < 0.1 indicates adequate balance between groups after propensity score matching.

## Data Availability

The data presented in this study are available on reasonable request from the corresponding author. Access is restricted in accordance with contractual agreements governing the use of the TMS registry. Data may be obtained either through the establishment of a formal data-use agreement or by institutional membership in the international TMS registry project, facilitated via the corresponding author.

## Supplementary Materials

The following supporting information can be downloaded at: https://www.mdpi.com/article/10.3390/biomedicines14081872/s1, Figure S1: Love Plot Demonstrating Covariate Balance Before and After Matching, Figure S2: Propensity Score Distribution and Overlap Between the Long COVID-Associated Depression Group and Primary MDD Group, Table S1: Sensitivity Analyses Using Alternative Propensity Score Matching Specifications.

## Abbreviations

The following abbreviations are used in this manuscript:

ACC	Anterior cingulate cortex
COVID-19	Coronavirus disease 2019
DLPFC	Dorsolateral prefrontal cortex
DSM-5	Diagnostic and Statistical Manual of Mental Disorders, Fifth Edition
EEG	Electroencephalography
HAM-D_17_	17-item Hamilton Depression Rating Scale
iTBS	Intermittent theta burst stimulation
IPTW	Inverse probability of treatment weighting
MADRS	Montgomery–Åsberg Depression Rating Scale
MDD	Major depressive disorder
RMT	Resting motor threshold
rTMS	Repetitive transcranial magnetic stimulation
SDM	Shared decision making
SMD	Standardized mean difference
TMS	Transcranial magnetic stimulation
TMS–EEG	Combined transcranial magnetic stimulation and electroencephalography
