# Supplementary material for: Comparative Treatment Response to Intermittent Theta Burst Stimulation in Long COVID-Associated Depression Versus Major Depressive Disorder: A Propensity Score-Matched Retrospective Cohort Study
Source: Biomedicines. 2026 Aug 21;14(8):1872. doi: 10.3390/biomedicines14081872 (PMC13509738; doi:10.3390/biomedicines14081872)
Supplement: Supplementary file 1 [file biomedicines-14-01872-s001.zip › biomedicines-4478903-supplementary.pdf]

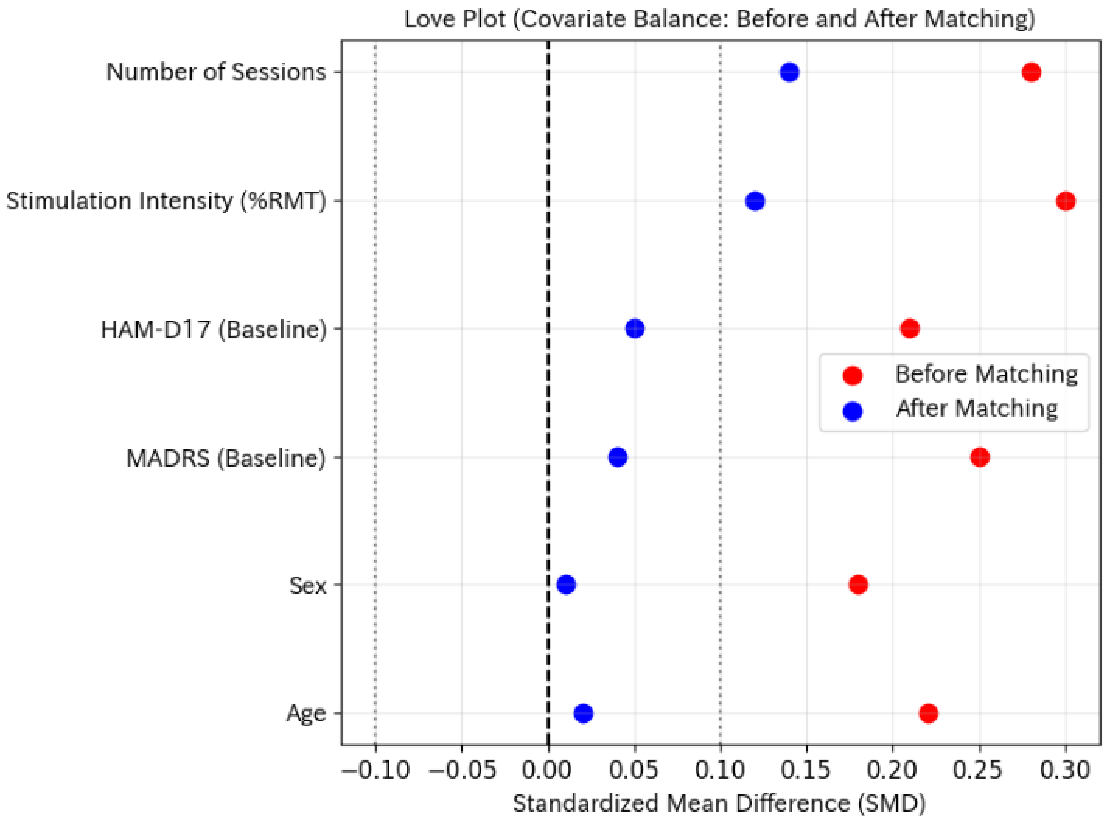

Figure S1. Love Plot Demonstrating Covariate Balance Before and After Matching

This Love plot illustrates standardized mean differences (SMDs) for all covariates before and after propensity score matching. Core demographic and baseline severity variables (age, sex, baseline MADRS, and baseline HAM-D_{17}) achieved SMD values below 0.1 following matching, indicating adequate balance between the Long COVID and primary MDD groups. Stimulation intensity and number of sessions exhibited minor residual imbalance (SMDs of 0.12 and 0.14), which were subsequently adjusted in inverse probability of treatment weighting (IPTW) and regression models. Overall, the plot demonstrates substantial improvement in covariate balance after matching, supporting the validity of the adjusted comparative analyses.

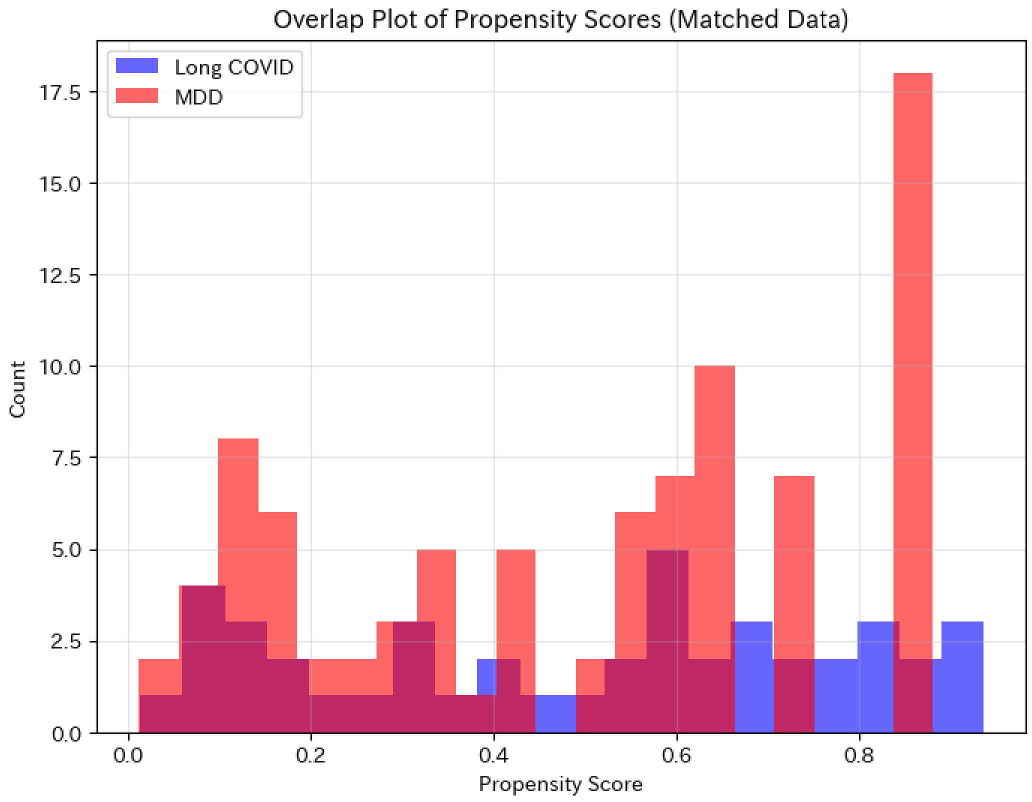

Figure S2. Propensity Score Distribution and Overlap Between the Long COVID-Associated Depression Group and Primary MDD Group

This figure illustrates the distribution of propensity scores for patients with Long COVID–associated depression and those with primary major depressive disorder (MDD) following 1:2 nearest-neighbor matching. The substantial overlap across the central range of the propensity score distribution indicates adequate common support between groups, supporting the validity of adjusted comparative analyses. Residual unmeasured confounding cannot be excluded due to the observational study design.

Table S1. Sensitivity Analyses Using Alternative Propensity Score Matching Specifications

Matching Specification	MADRS Improvement Difference (percentage points)	HAM-D$_{17}$ Improvement Difference (percentage points)
**Nearest-neighbor matching (without replacement)**	−18.57	−17.02
**Caliper-restricted matching (0.20 SD of logit PS)**	−18.04	−17.18
**Matching including baseline HAM-D17**	−9.97	−11.83

Note: Negative values indicate lower improvement in the Long COVID group relative to the matched primary MDD group. All specifications demonstrated consistent directionality, supporting the robustness of the observed adjusted associations.
